# Differences in meat quality between Angus cattle and Xinjiang brown cattle in association with gut microbiota and its lipid metabolism

**DOI:** 10.3389/fmicb.2022.988984

**Published:** 2022-12-06

**Authors:** Zhuo Chen, Yawei Sun, Lijing Chen, Yang Zhang, Jinquan Wang, Hongbo Li, Xiangming Yan, Lining Xia, Gang Yao

**Affiliations:** ^1^College of Veterinary Medicine, Xinjiang Agricultural University, Urumqi, China; ^2^Institute of Animal Science, Xinjiang Academy of Animal Sciences, Urumqi, China; ^3^Xinjiang Key Laboratory of New Drug Study and Creation for Herbivorous Animals (XJKLNDSCHA), Xinjiang Agricultural University, Urumqi, China

**Keywords:** Xinjiang brown cattle, Angus cattle, meat quality traits, lipid metabolism, gut microbiota, metabolomics

## Abstract

Gut microbiota plays important roles in mediating fat metabolic events in humans and animals. However, the differences of meat quality traits related to the lipid metabolism (MQT-LM) in association with gut microbiota involving in lipid metabolism have not been well explored between Angus cattle (AG) and Xinjiang brown cattle (BC). Ten heads of 18-month-old uncastrated male AG and BC (5 in each group) raised under the identical conditions were selected to test MQT-LM, i.e., the backfat thickness (BFT), the intramuscular fat (IMF) content, the intramuscular adipocyte areas (IAA), the eye muscle area (EMA), the muscle fiber sectional area (MFSA) and the muscle shear force after sacrifice. The gut microbiota composition and structure with its metabolic function were analyzed by means of metagenomics and metabolomics with rectal feces. The correlation of MQT-LM with the gut microbiota and its metabolites was analyzed. In comparison with AG, BC had significant lower EMA, IMF content and IAA but higher BFT and MFSA. Chao1 and ACE indexes of α-diversity were lower. β-diversity between AG and BC were significantly different. The relative abundance of Bacteroidetes, *Prevotella* and *Blautia* and *Prevotella copri*, *Blautia wexlerae*, and *Ruminococcus gnavus* was lower. The lipid metabolism related metabolites, i.e., succinate, oxoglutaric acid, L-aspartic acid and L-glutamic acid were lower, while GABA, L-asparagine and fumaric acid were higher. IMF was positively correlated with *Prevotella copri*, *Blautia wexlerae* and *Ruminococcus gnavus*, and the metabolites succinate, oxoglutaric acid, L-aspartic acid and L-glutamic acid, while negatively with GABA, L-asparagine and fumaric acid. BFT was negatively correlated with *Blautia wexlerae* and the metabolites succinate, L-aspartic acid and L-glutamic acid, while positively with GABA, L-asparagine and fumaric acid. *Prevotella Copri*, *Blautia wexlerae*, and *Ruminococcus gnavus* was all positively correlated with succinate, oxoglutaric acid, while negatively with L-asparagine and fumaric acid. In conclusion, *Prevotella copri*, *Prevotella intermedia*, *Blautia wexlerae*, and *Ruminococcus gnavus* may serve as the potential differentiated bacterial species in association with MQT-LM *via* their metabolites of oxoglutaric acid, succinate, fumaric acid, L-aspartic acid, L-asparagine, L-glutamic acid and GABA between BC and AG.

## Introduction

Beef is a main source of animal protein in human food, and consumers are paying an increasing amount of attention to meat quality. Most of meat quality traits that are closely related to lipid metabolism (MQT-LM) include the backfat thickness (BFT), intramuscular fat (IMF) content, the intramuscular adipocyte areas (IAA), the eye muscle area (EMA) and the muscle fiber sectional area (MFSA), marbling and tenderness, they are mainly involved in the fat metabolism/deposition in the beef (Picard et al., 2018; Li et al., 2020). Although a large number of studies on MQT-LM have been performed in Angus cattle (AG), few studies have systematically compared MQT-LM in AG with Xinjiang brown cattle (BC), an indigenous dual- purpose of meat and milk cattle breed with excellent adaptability, strong disease resistance, good grazing and tolerance to extreme weather in China (Li et al., 2020). Therefore, the comparison on the difference of meat quality between AG and BC is pivotal for assisting the improvement of meat quality of Xinjiang brown cattle.

The gut microbiota is a highly complex and diverse microecosystem which is strongly associated with various host physiological functions, e.g., metabolism, immune regulation, growth and development and production performance, and to overall host health and disease in human and animals (Turnbaugh and Gordon, 2009; Petri et al., 2013; Lee and Hase, 2014; Myer et al., 2015; Noel et al., 2019). Large body of studies have figured out the contribution of microbiota on host health and indicated marked shifts in the relative abundance of various phyla or species in various physiological statuses (Li et al., 2017; Franzosa et al., 2019; Wu et al., 2020). Regarding the gut microbiota in the interaction with host lipid metabolism and body fat deposition, most studies are human or experimental rodents orientated for human health. For example, the comparisons of the gut microbiota of lean and obese people indicated that the relative abundance of Bacteroidetes and Firmicutes are associated with obesity (Ley et al., 2005, 2006). Additionally, gut microbiota may directly regulate the expression of genes related to fatty acid synthesis and triglyceride storage in animals, thus altering energy balance to determine adipose tissue expansion, or fat deposition, in different parts of the body (Hooper et al., 2001). When gut microbiota obtained from obese mice are transplanted into non-obese, aseptic mice, the previously non-obese mice exhibit greater efficiency in energy absorption in the gut and significant increases in total body fat (Turnbaugh et al., 2006). Bäckhed et al. (2004) experiment showed that the gut microbiota acts through angiopoietin-like protein 4 as to coordinate increased hepatic lipogenesis with increased lipoprotein lipase activity in adipocytes, thereby promoting storage of calories harvested from the diet into fat. In terms of gut microbiota and its lipid metabolic regulating function on MQT-LM / fat deposition in food animals, there are already some studies in pigs (Khanal et al., 2020; Wu et al., 2021; Ma et al., 2022), chickens or broilers (Wen et al., 2019; Yang et al., 2021), and ducks (Lyu et al., 2021). However, the association of MQT-LM with the gut microbiota and its lipid metabolic functions on beef cattle has been seldom elucidated. Zhang and colleagues did the comparison of the gut microbiota in Angus beef cattle reared under the grazing and feedlot conditions and then speculated that the significant differences in gut microbiota composition may have an impact on the meat quality (Zhang et al., 2021). Zheng et al. (2022) studied the association of gut microbiota with host intramuscular differentially expressed genes and metabolites in Angus and Chinese Simmental cattle, unveiled the different associations of gut microbiota and the meat quality between these two breeds. Therefore, we hypothesized that differentials MQT-LM between different AG and BC could be associated with the specific composition and structure in gut microbiota and its metabolic function. MQT-LM namely, the BFT, IMF, IAA, EMA, and MFSA were assessed using 18-month-old BC and AG. The structure, function and metabolic pathways of the gut microbiota were determined by metagenomics and metabolomics and their associations with meat quality traits were determined by correlation analysis so as to provide a potential novel biomarker for improving the meat quality of BC.

## Materials and methods

### Animals, housing, and feeding

Eighteen-month-old uncastrated male AG (*n* = 5) and BC (*n* = 5), which were collected at age of about 5-month-old with initial body weight between 170 and 180 kg from a beef cattle breeding farm in Xinjiang, raised under the identical feeding regime, management and condition (Table 1) were used for the study. The study protocol was approved by the Animal Ethics Committee of Xinjiang Agricultural University (2017015).

**Table 1:** Diet composition and nutrient levels (based on dry matter).

Feed ingredient	Weight (kg)	Content (%)	Nutritional ingredient	Content
Straws	1.3	5.78	Metabolic energy (MJ/kg)	5.82
Alfalfas	0.5	2.22	Crude protein (%)	16.2
Cossettes	2	8.89	Crude fat (%)	2.32
Wheat straws	1.5	6.67	Calcium (%)	0.54
Ensilings	10	44.46	Phosphorus (%)	0.49
Molasses	0.7	3.11	Acid detergent fiber (%)	5.9
Concentrates*	6.49	28.86	Neutral detergent fiber (%)	12.98
Total	22.49	100	Total digestible nutrient (%)	61.57

* Concentrate: corn (53.16%), cotton meal (11.56%), bran (7.40%), magnesium oxide (3.08%), protein feed (23.42) and premix (1.38%) containing (vitamin A, vitamin B1, vitamin B2, vitamin B6, vitamin D, vitamin E, pantothenic acid, nicotinamide, Cu, Fe, Mn, Zn, Se, and Co).

### Sample collection

Bulls were fasted for 24 h, but provided water *ad libitum* up 12 h prior to sacrifice. Following the 24 h fast, cattle were weighed. About 20 g of feces was collected from the rectum of cattle by samplers equipped with aseptic gloves 6 h prior to slaughter, and then placed in a sterile, frozen tube and quickly stored in liquid nitrogen for subsequent testing. All the bulls were slaughtered in the automatic cattle slaughter line of a slaughterhouse head by head in the same day. The bulls’ carcass was weighed after sacrifice, and the *longissimus dorsi* muscle was dissected, about 1 cm^3^ sample of it was collected and fixed in picric acid solution, the remaining muscle was stored at 4°C for shear force examination.

### Meat quality traits detection

The BFT and the EMA were measured *in vivo* by veterinary B-ultrasound (Pyle Co. LTD, Aquila Vet, Netherlands) *via* detector located on the back of *longissimus dorsi* muscle between intercostal space of 12th and 13th ribs. The muscle shear force was measured using a computer-coupled muscle tenderness instrument (Brad Technology Development Co. Ltd., c-lm4, Beijing, China) as described elsewhere (Wen et al., 2020; Bai et al., 2022). The muscle moisture content was measured by the oven drying method, the samples were dried at 101–105°C for 24 h under the constant temperature and pressure condition. The moisture (%) was calculated by calculation formula: (sample weight before drying – weight after drying) / weight before drying × 100 (Mamani-Linares and Gallo, 2013; Wen et al., 2020). The ash content was measured by high temperature burning method, the samples were burned in furnace at ca 500°C. The ash (%) was calculated by calculation formula: (sample weight before burning – weight after burning) / weight before burning × 100 (Latimer, 2012; Luo et al., 2019). IMF was determined by Soxhlet extraction, in which 2.5 g of freeze-dried *longissimus dorsi* muscle was extracted in 85 ml of hexane for 60 min and then placed in a forced draft oven for 30 min at 105°C. The variation in sample weight before and after extraction was used to calculate fat content (Holman et al., 2019; Chen G. et al., 2021). Fixed muscle tissue was dehydrated and paraffin-embedded, paraffin sections were prepared, stained with oil red O, and observed under an optical microscope (Nikon Instruments Co. Ltd., 55I-1,000, Shanghai, China). Adipocyte diameter was measured using a microscopic imaging system (Motic Advanced 3.5). Three non-consecutive muscle sections from each muscle sample were used to measure adipocyte diameter. Within each section, adipocyte diameter was measured in three separate field of vision. All measurements for each bull were averaged and used for statistical analyses. The IAA and the MFSA were measured and calculated (Pertl et al., 2013; Ding et al., 2021).

### Metagenomics of gut microbiota

Total DNA was extracted from cattles’ feces with OMEGA Soil DNA Kit (D5625-01), as previously described by Huang et al. (2019). DNA concentration and quality were determined by NanoDropND-1,000 spectrophotometer (Thermo Fisher Scientific, Waltham, MA, United States) and agarose gel electrophoresis, respectively. The extracted microbial DNA was processed to construct metagenome shotgun sequencing libraries with insert sizes of 400 bp by using the Illumina TruSeq Nano DNA LT Library Preparation Kit. Each library was sequenced by the Illumina HiSeq X-ten platform (Illumina, United States) with PE150 strategy.

Raw sequencing reads were processed to obtain quality-filtered reads for further analysis. Adapter sequences were removed by Cutadapt (v1.2.1; Marcel, 2011). The sequencing reads were aligned to the host genome using BWA to remove host contamination. Quality-filtered reads were *de novo* assembled to construct the metagenome for each sample by IDBA-UD (Iterative De Bruijn graph Assembler for sequencing data with highly Uneven Depth). The coding sequences (CDS, > 300 bp) were predicted by MetaGeneMark (v3.25; Zhu et al., 2010). CDSs were clustered by CD-HIT (v4.8.1) at 90% amino acid sequence identity to obtain a non-redundant gene catalogue. The alpha diversity index (Chao1 index, Ace index, Simpson index, and Shannon index) was based on Mothur (version 1.30.1). The beta-diversity of both bacterial and fungal communities was assessed by computing weighted UniFrac distance matrices and then ordinated using non-metric multi-dimensional scaling (NMDS). The relative contribution of different biotic and abiotic factors on community dissimilarity was tested with PERMANOVA using the Adonis function. Linear discriminant analysis Effect Size (LEfSe) was measured the consistency of differences in relative abundance between taxa in the groups analyzed (BC vs. AG), taxa with LDA score > 2 and *p* value <0.05 set as the significant level. Gene abundance in each sample was estimated by SOAPdenovo2 (v1.0). The taxonomy was annotated by searching against the NCBI-NT database by BLASTN (*e* value <0.001) and annotated by MEGAN with the lowest common ancestor approach. The functional gene was annotated by searching the sequence of the non-redundant genes against the KEGG databases (release 90.0) by DIAMOND protein aligner (v2.0.4; Cantalapiedra et al., 2021). Gene abundances were derived from mapping the all reads back to the predicted ORF *via* bowtie2 (v2.2.6) and calculated transcripts per kilobase million (TPM) *via* SamTools (v1.5; Rausch et al., 2019). Metagenomic sequencing were analyzed at Personal Biotechnology Co., Ltd. (Shanghai, China).

### Metabolomics analysis

LC–MS was performed as previously described (Fan et al., 2021). For each fecal sample, 100 mg was transferred into a 2 ml centrifuge tube and 500 μl of ddH2O at 4°C added. The supernatant was collected by centrifugation. Chromatographic separation was accomplished in an Thermo Ultimate 3,000 system equipped with an ACQUITY UPLC® HSS T3 (150 × 2.1 mm, 1.8 μm, Waters) column maintained at 40°C. The temperature of the autosampler was 8°C. Gradient elution of analytes was carried out with 0.1% formic acid in water (C) and 0.1% formic acid in acetonitrile (D) or 5 mM ammonium formate in water (A) and acetonitrile (B) at a flow rate of 0.25 ml/min. Injection of 2 μl of each sample was done after equilibration. Analytes from fecal samples were obtained *via* chromatographic separation and subjected to electrospray ionization multistage mass spectrometry (ESI-MSn) on the Thermo Q Exactive mass spectrometer. LC–MS was performed by Personal Biotechnology Co., Ltd. (Shanghai, China).

### Statistical analysis

Inter-group variables were analyzed by Mann–Whitney test using GraphPad Prism 9.0.0 (GraphPad Software, San Diego, CA, United States). Data are reported as mean ± SEM. * denotes *p* < 0.05, meaning significant difference, ** denotes *p* < 0.01, meaning extremely significant difference. The differential species in the gut microbiota between BC and AG screened by metagenomics analysis were correlated with the differential MQT-LM and metabolites, separately, and then the correlation of MQT-LM with metabolites was analyzed by Spearman correlation method. Based on R (Version 3.6.2), ggplot2 and corrplot packages were used for correlation analysis and mapping. Spearman correlation coefficient was calculated, and significance test was conducted.

## Results

### Meat quality detection

**Figure 1:**
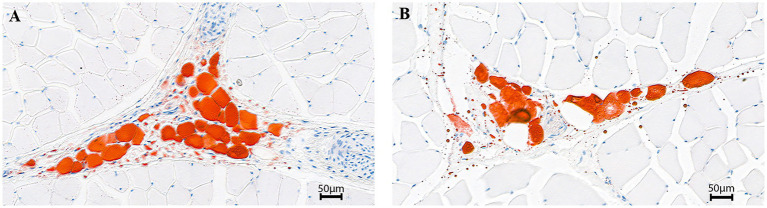
Intramuscular fat in longissimus dorsi sections (200×). Angus cattle **(A)**; Xinjiang brown cattle **(B)**.

The body and carcass weight of BC was extremely lower than that of AG (*p* < 0.01). The EMA, IMF content, muscle ash content and IAA were significantly lower in BC (*p* < 0.05), however, the BFT and MFSA were significantly higher in BC (*p* < 0.05; Figure 1; Table 2).

**Table 2:** Comparison of beef quality traits.

Characteristics	AG	BC	*p* value
Weight, kg	625.39 ± 21.51	482.65 ± 19.16	0.001
Carcass weight, kg	379.92 ± 17.74	278.73 ± 6.88	0.001
Backfat thickness (BFT), mm	5.76 ± 0.27	6.62 ± 0.21	0.023
Eye muscle area (EMA), cm^2^	80.23 ± 2.78	71.05 ± 2.91	0.035
Intramuscular fat (IMF), %	11.09 ± 0.39	9.74 ± 0.43	0.032
Muscle shear force (MSF), N	46.35 ± 6.33	46.07 ± 6.19	0.972
Moisture content (MC), %	71.28 ± 0.78	72.71 ± 1.27	0.348
Ash content (AC), %	7.56 ± 0.31	6.58 ± 0.29	0.032
Muscle fiber sectional area (MFSA), μm^2^	1248.39 ± 154.58	1662.83 ± 65.26	0.039
Intramuscular adipocyte areas (IAA), μm^2^	48310.08 ± 9752.09	18312.65 ± 2721.01	0.018

### Metagenomics of gut microbiota

#### Composition analysis of gut microbiota

**Figure 2:**
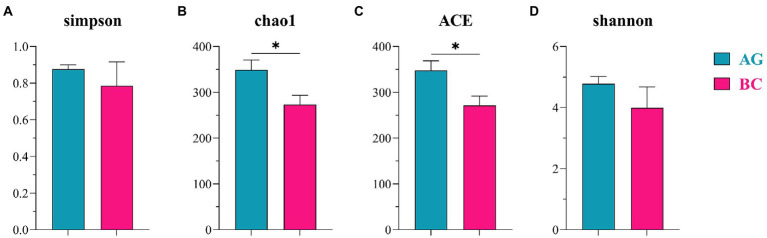
Comparison of α-diversity in the gut microbiota between Xinjiang brown cattle (BC) and Angus cattle (AG). (*n* = 5 in each breed). Simpson diversity index **(A)**; The Chao1 estimator **(B)**; The ACE estimator **(C)**; Shannon diversity index **(D)**. Values were expressed as means ± SEM. * denotes *P* < 0.05 indicating significant difference.

**Figure 3:**
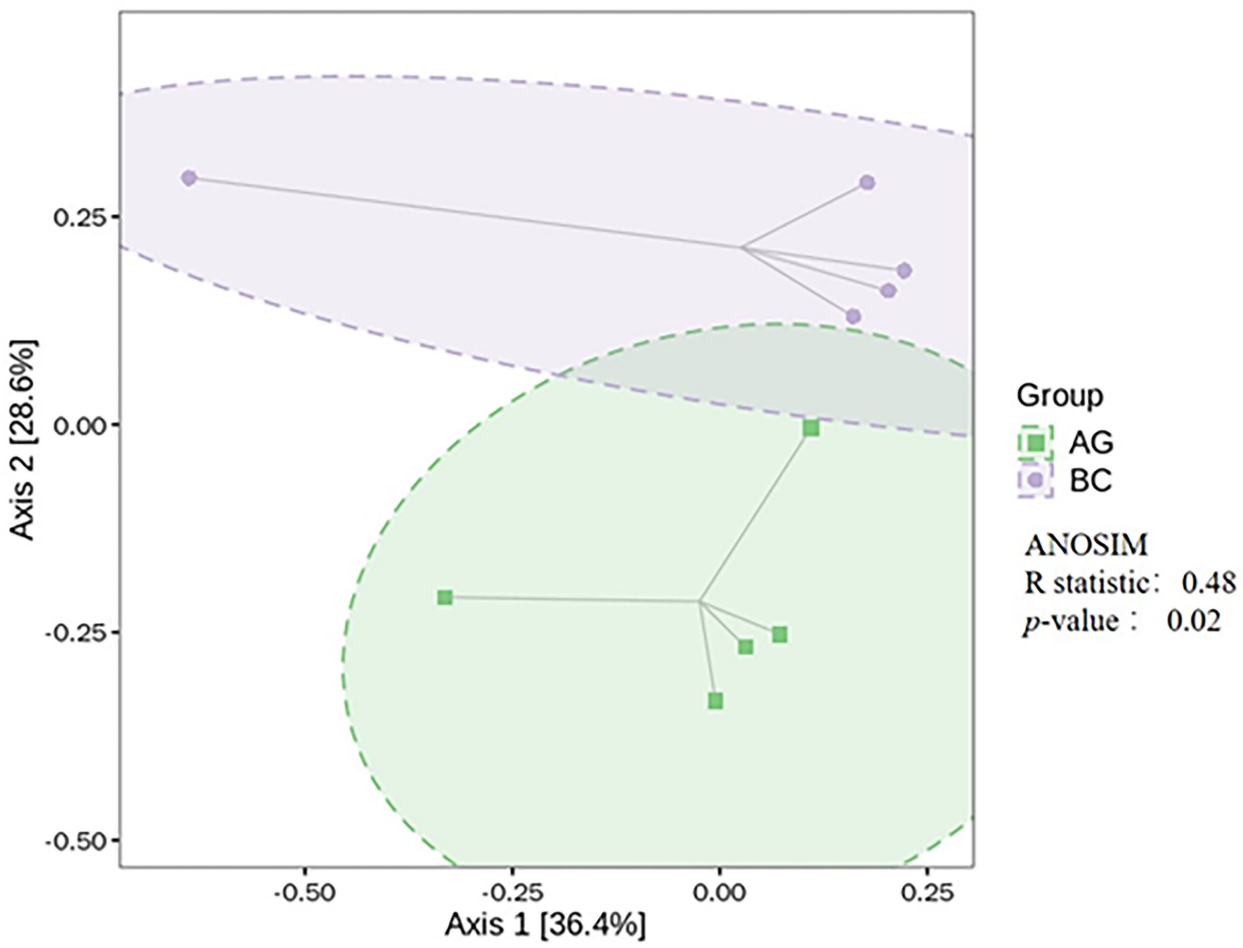
Comparison of β-diversity in the gut microbiota between Xinjiang brown cattle (BC) and Angus cattle (AG). (*n* = 5 in each breed). ANOSIM, analysis of similarities.

The α-diversity indices of gut microbiota (Simpson, Chao1, ACE, Shannon) and β-diversity were compared in BC and AG shown in Figures 2, 3, respectively. Chao1 and ACE in BC were significantly lower than those in AG (*p* < 0.05). The β-diversity of gut microbiota showed extremely significant independent distribution between AG and BC (Anosim *p* < 0.01).

**Figure 4:**
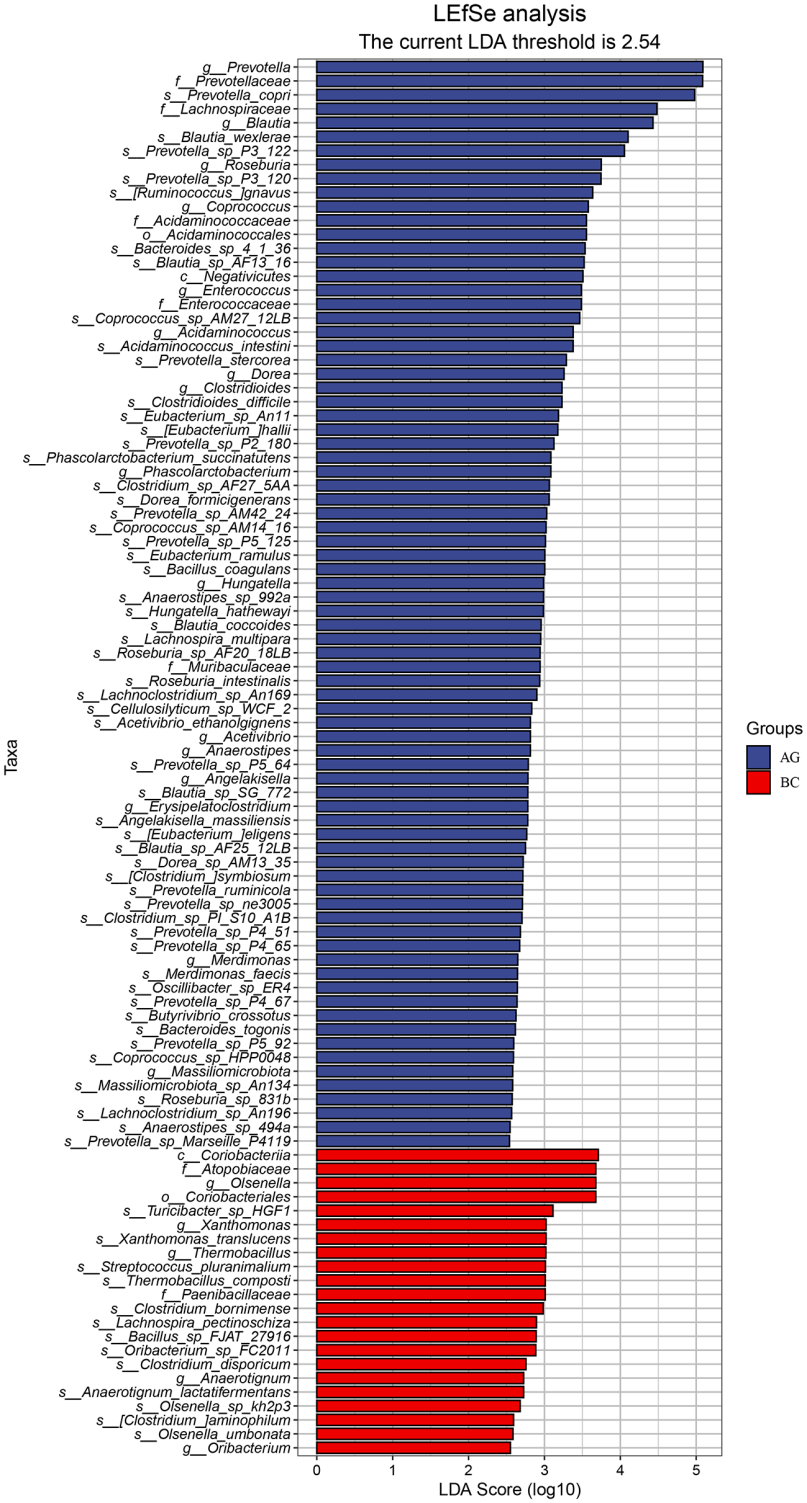
LEfSe analysis in the gut microbiota between Xinjiang brown cattle (BC) and Angus cattle (AG). (*n* = 5 in each breed). LEfSe, Linear discriminant analysis Effect Size.

**Figure 5:**
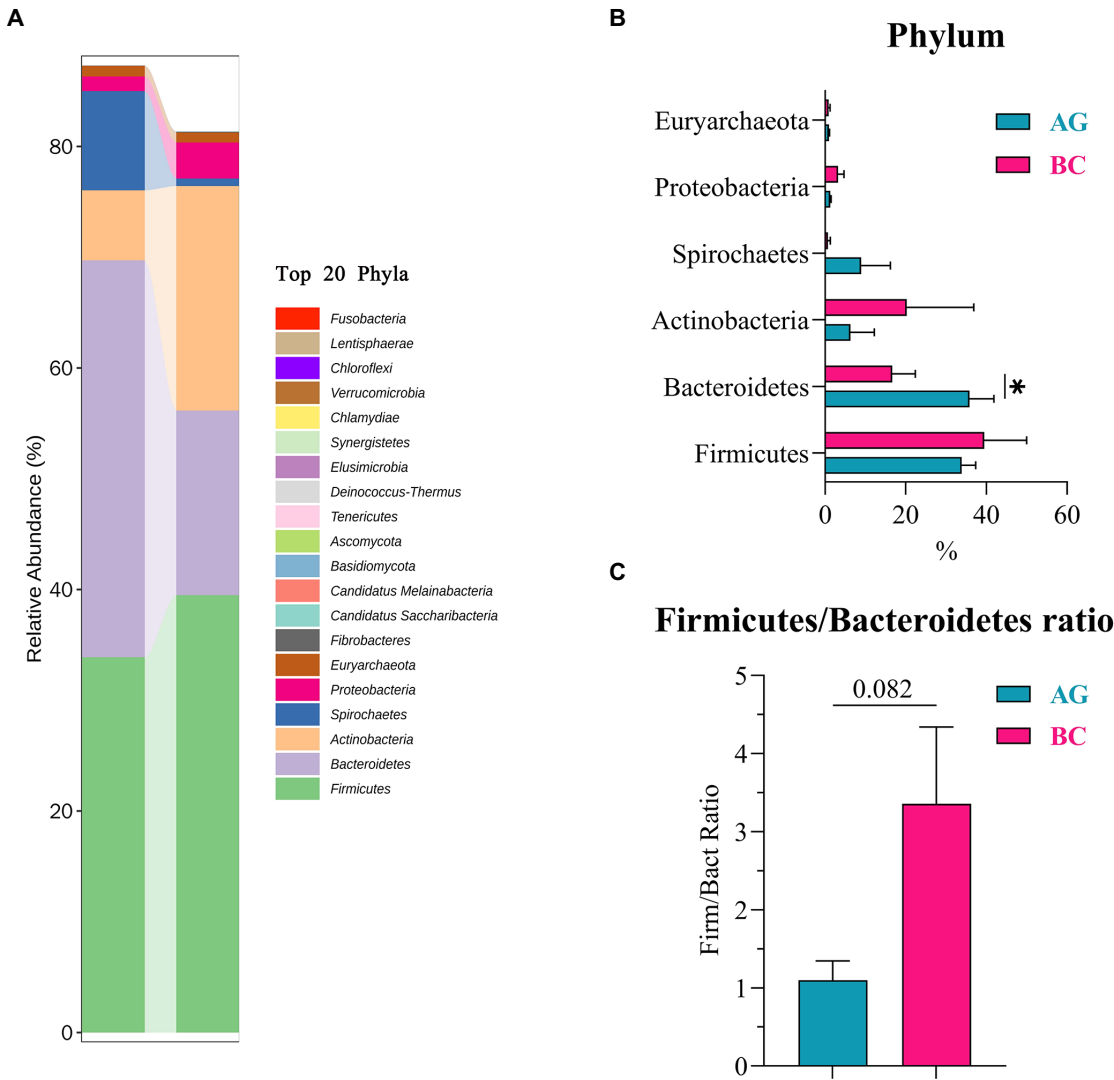
The phyla composition and the differentials in the gut microbiota between Xinjiang brown cattle (BC) and Angus cattle (AG; top 20 Phyla). (*n* = 5 in each breed). The relative abundance of phyla composition **(A)**; The differential phyla in the different relative abundance **(B)**; Firmicutes/Bacteroidetes Ratio **(C)**. Values were expressed as means ± SEM. * denotes *p* < 0.05 indicating significant difference.

**Figure 6:**
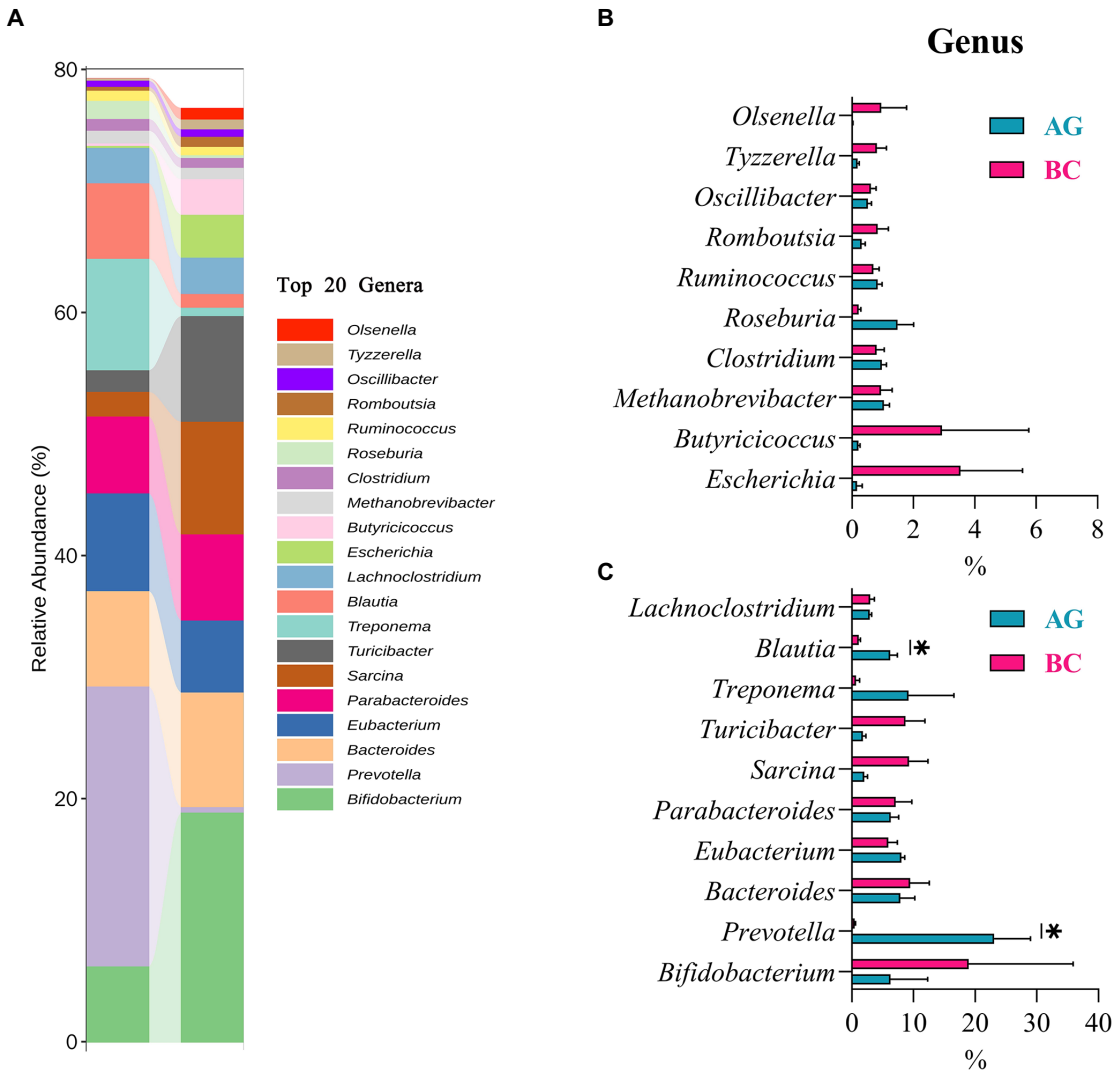
The genera composition and the differentials in the gut microbiota between Xinjiang brown cattle (BC) and Angus cattle (AG; top 20 genera). (*n* = 5 in each breed). The relative abundance of genera composition **(A)**; The differential genera in the different relative abundance **(B,C)**. Values were expressed as means ± SEM. * denotes *p* < 0.05 indicating significant difference.

**Figure 7:**
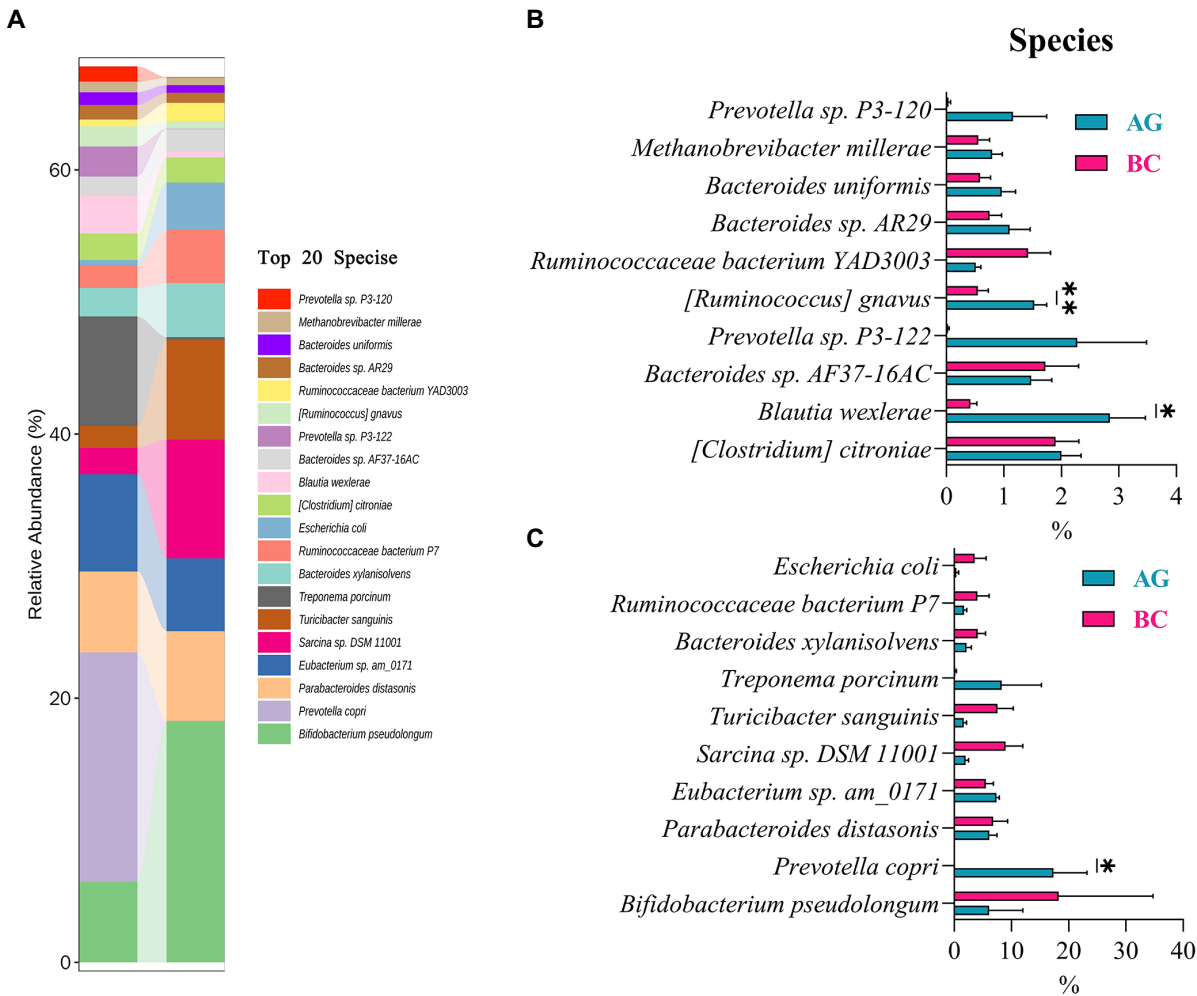
The species composition and the differentials in the gut microbiota between Xinjiang brown cattle (BC) and Angus cattle (AG; top 20 species). (*n* = 5 in each breed). The relative abundance of species composition **(A)**; The differential species in the different relative abundance **(B,C)**. Values were expressed as means ± SEM. * denotes *p* < 0.05 indicating significant difference, ** denotes *p* < 0.01 indicating extremely significant difference.

Results of linear discriminant analysis effect size (LEfSe) showed the gut microbiota was significantly different between AG and BC (Figure 4). At the class level, the Coriobacteriia was enriched in BC and Negativicutes enriched in AG. At the order level, the Coriobacteriales was enriched in BC and Acidaminococcales enriched in AG. At the family level, there were 7 bacteria in gut microbiota significantly different between BC and AG, in which 2 families were enriched in BC and 5 families enriched in AG. At the genus level, there were 21 bacteria significantly different in gut microbiota between BC and AG, in which 5 genera were enriched in BC and 16 genera enriched in AG. At the species level, there were 68 bacteria significantly different in gut microbiota between BC and AG, in which 13 species were enriched in BC and 55 species enriched in AG (Figure 4). As shown in Figure 5A, in both BC and AG gut microbiota were mainly comprised of bacteria within phyla of Firmicutes, Bacteroidetes, Actinobacteria, and Spirochaetes with the relative abundance of Firmicutes and Bacteroidetes being highest. The relative abundance of Bacteroidetes in BC was significantly lower than that in AG (*p* < 0.05; Figure 5B) thus resulting in the ratio of Firmicutes/Bacteroidetes in the relative abundance had a tendency of increase in BC (*p* = 0.08; Figure 5C). *Bifidobacterium*, *Prevotella*, *Bacteroides* and *Eubacterium* were the most abundant genera in both BC and AG (Figure 6A). The relative abundance of *Prevotella* and *Blautia* were significantly lower in BC if compared to AG (*p* < 0.05; Figures 6B,C). The top 20 species in the gut microbiota between breeds were listed in Figure 7A with the highest relative abundance of *Prevotella copri* in AG and *Bifidobacterium pseudolongum* in BC (Figure 7A). The relative abundance of *Prevotella copri* and *Blautia wexlerae* in BC were significantly lower than that in AG (*p* < 0.05; Figures 7B,C). The relative abundance of *Ruminococcus gnavus* was extremely lower in BC than that in AG (*p* < 0.01; Figures 7B,C).

#### Functional analysis of gut microbiota

**Figure 8:**
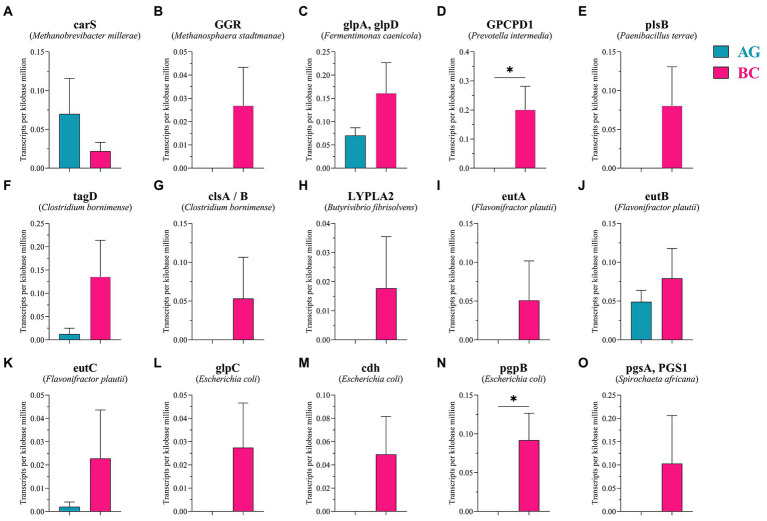
The relative abundance of genes related to lipid metabolism in lipid metabolism pathways between Xinjiang brown cattle (BC) and Angus cattle (AG). (*n* = 5 in each breed). The expression of genes related to lipid metabolism **(A–O)**. Values were expressed as means ± SEM. * denotes *p* < 0.05 indicating significant difference.

Protein sequences were annotated using the KEGG database, and the number of KEGG metabolic pathways annotated to different grades and categories was determined according to their KEGG Orthology (KO) classification. As shown in Supplementary Figure 1, enriched KEGG pathways within metabolism function included: carbohydrate metabolism, amino acid metabolism, nucleotide metabolism and lipid metabolism. Protein sequences and KO data were obtained from the gene coding regions of gut microbiota (Table 3). Sixteen functional genes related to lipid metabolism were identified within the KEGG differential lipid metabolism pathway (Pathway ID: ko00564) and enriched in 10 bacterial species: *Methanobrevibacter millerae*, *Methanosphaera stadtmanae*, *Fermentimonas caenicola*, *Prevotella intermedia*, *Paenibacillus terrae*, *Clostridium bornimense*, *Butyrivibrio fibrisolvens*, *Flavonifractor plautii*, *Escherichia coli*, and *Spirochaeta africana*. TPM values of lipid metabolism related functional gene pgpB derived from *Escherichia coli* and GPCPD1 from *Prevotella intermedia* were significantly higher in the gut microbiota of BC than those in the gut microbiota of AG (*p* < 0.05; Figure 8; Table 3).

**Table 3:** Genes of the glycerophospholipid metabolism pathway.

Protein ID	KO	Gene name	Pathway ID	Taxonomy
gene_9708818	K19664	carS	ko00564	*Methanobrevibacter millerae*
gene_9222867	K17830	GGR	ko00564	*Methanosphaera stadtmanae*
gene_9997979	K00111	glpA, glpD	ko00564	*Fermentimonas caenicola*
gene_8259284	K18695	GPCPD1	ko00564	*Prevotella intermedia*
gene_9671445	K00631	plsB	ko00564	*Paenibacillus terrae*
gene_9906485	K00980	tagD	ko00564	*Clostridium bornimense*
gene_9981399	K06131	ClsA/B	ko00564	*Clostridium bornimense*
gene_7283784	K06130	LYPLA2	ko00564	*Butyrivibrio fibrisolvens*
gene_9986649	K04019	eutA	ko00564	*Flavonifractor plautii*
gene_9986650	K03735	eutB	ko00564	*Flavonifractor plautii*
gene_9986651	K03736	eutC	ko00564	*Flavonifractor plautii*
gene_9947823	K00113	glpC	ko00564	*Escherichia coli*
gene_12284373	K01521	cdh	ko00564	*Escherichia coli*
gene_8324574	K01096	pgpB	ko00564	*Escherichia coli*
gene_9999662	K00995	pgsA, PGS1	ko00564	*Spirochaeta africana*

### Metabolomics of gut microbiota

**Figure 9:**
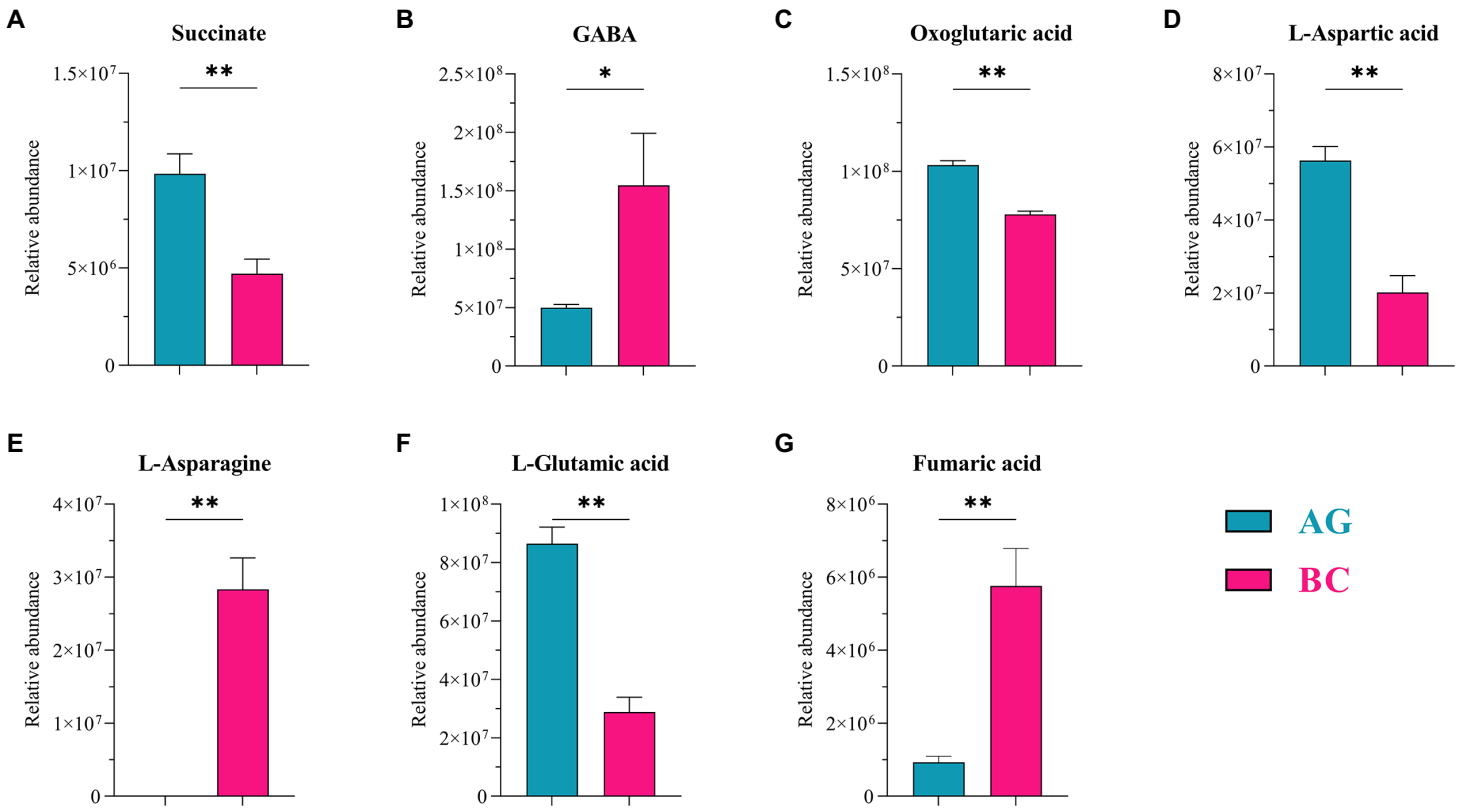
Metabolites in gut microbiota related to lipid metabolism between Xinjiang brown cattle (BC) and Angus cattle (AG). (*n* = 5 in each breed). The bacterial metabolites related to lipid metabolism **(A–G)**. Values were expressed as means ± SEM. * denotes *p* < 0.05 indicating significant difference, ** denotes *p* < 0.01 indicating extremely significant difference.

Metabolomic analysis identified 450 microbiota-derived metabolites that differed between Xinjiang brown and AG (Supplementary Figure 2). These included mainly carboxylic acids and derivatives (15.66%), fatty acyls (14.46%), benzene and substituted derivatives (11.65%), organooxygen compounds (6.43%) and steroids and steroid derivatives (5.02%; Supplementary Figure 2). Two metabolic pathways related to lipid metabolism, i.e., D-glutamine and D-glutamate metabolism and Alanine, aspartate and glutamate metabolism, were used in subsequent analyses based on pathway impact analysis (Supplementary Figure 3). Within the enriched pathways, 7 microbiota-related metabolites were identified as being significantly different between BC and AG. These included oxoglutaric acid, succinate, fumaric acid, L-aspartic acid, L-asparagine, L-glutamic acid and GABA (Table 4). As shown in Figure 9, L-asparagine, fumaric acid (*p* < 0.01) and GABA (*p* < 0.05) were significantly higher, whereas succinate, oxoglutaric acid, L-aspartic acid and L-glutamic acid were significantly lower in BC (*p* < 0.01).

**Table 4:** Pathways enriched in microbiota-related metabolites.

Pathway	Compounds
Alanine, aspartate and glutamate metabolism (Total: 23; Hits: 7; Raw p: 0.01; −log(p): 4.52; Holm adjust: 0.88; FDR: 0.88; Impact: 0.63)	Fumarate acid
	Succinate acid
	Oxoglutaric acid
	L-asparagine
	L-Aspartic acid
	L-glutamate acid
	GABA
D-Glutamine and D-glutamate metabolism (Total: 5; Hits: 2; Raw p: 0.15; −log(p): 1.89; Holm adjust: 1.00; FDR: 1.00; Impact: 1.00)	L-glutamate acid
	Oxoglutaric acid

### Correlation analysis

**Figure 10:**
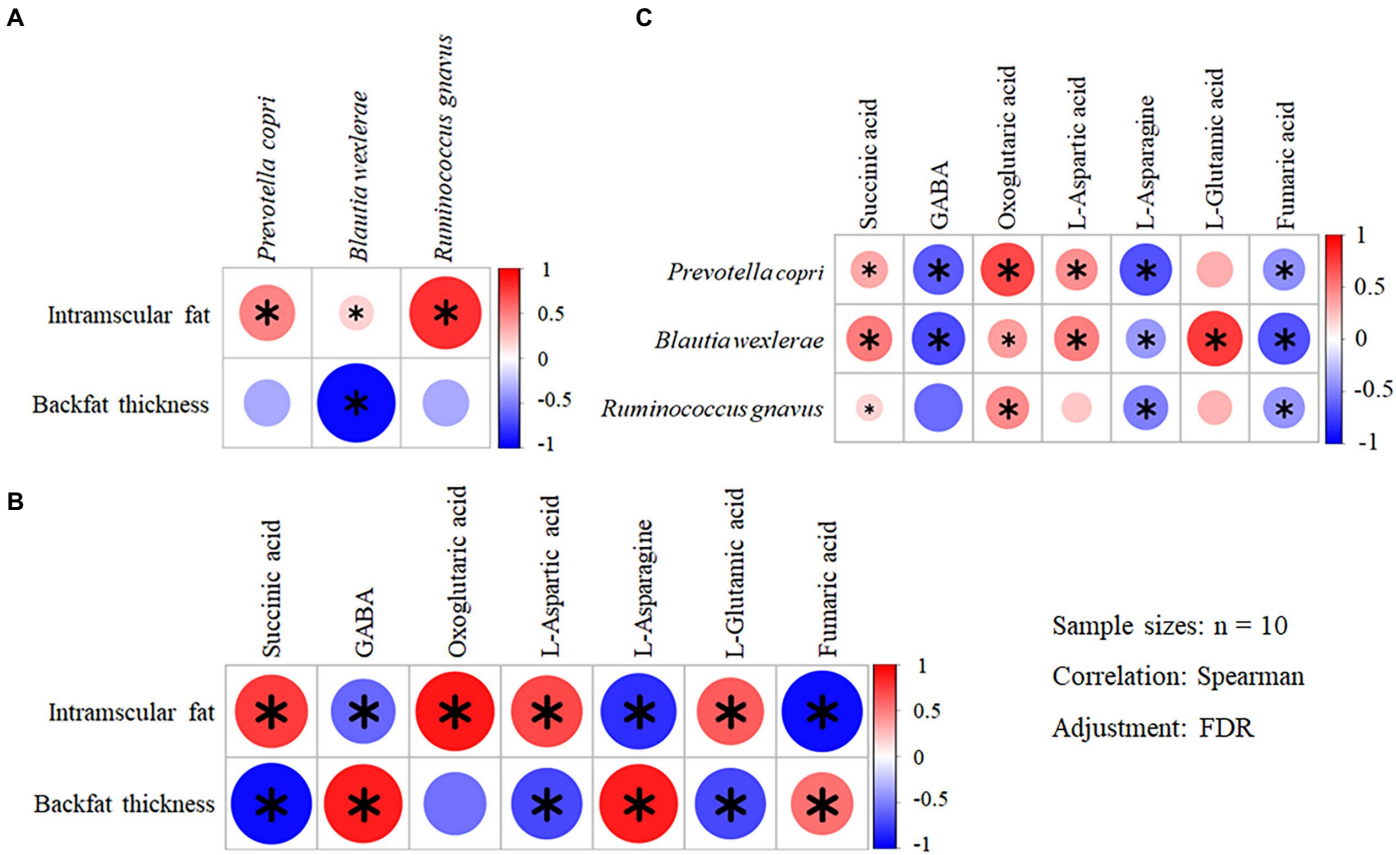
Correlation analyses of the differential MQT-LM – species – metabolites in Xinjiang brown cattle (BC) and Angus cattle (AG). (*n* = 10). Correlation of the differential MQT-LM and species **(A)**; Correlation of the differential MQT-LM and metabolites **(B)**; Correlation of the differential species and metabolites **(C)**. * denotes *p* < 0.05 indicating significant difference.

In total there were 76 species with the relative abundance above 0.1% (RA abv. 0.1%) in metagenomic analysis. They were all analyzed in association with 7 metabolites related to lipid metabolism and 10 MQT-LM by Spearman correlation. Among them, 8 species have different correlations with meat quality traits (Supplementary Figure 4A), 7 metabolites have different correlation with meat quality traits (Supplementary Figure 4B) and 12 species have different correlation with 7 metabolites related to lipid metabolism (Supplementary Figure 4C). Based on this result, the differential genus and species, the differential MQT-LM and the differential metabolites related to fat metabolism between AG and BC were further analyzed by Spearman correlation. IMF was positively correlated with *Prevotella copri* (*p* < 0.01, *r* = 0.84), *Blautia wexlerae* (*p* = 0.01, r = 0.78) and *Ruminococcus gnavus* (*p* < 0.01, *r* = 0.93; Figure 10A). BFT was negatively correlated with *Blautia wexlerae* (*p* < 0.01, *r* = −0.83; Figure 10A). The Spearman correlation analysis of MQT-LM and differential microbiota-related metabolites related to lipid metabolism showed that IMF was positively correlated with levels of succinate (*p* = 0.01, *r* = 0.81), oxoglutaric acid (*p* < 0.01, *r* = 0.83) and L-aspartic acid (*p* = 0.03, *r* = 0.71), and negatively correlated with levels of GABA (*p* = 0.03, *r* = −0.70), L-asparagine (*p* < 0.01, *r* = −0.85) and fumaric acid (*p* < 0.01, *r* = −0.87; Figure 10B). BFT was positively correlated with GABA (*p* = 0.01, *r* = 0.79), L-asparagine (*p* = 0.01, *r* = 0.80) and fumaric acid (*p* = 0.04, *r* = 0.66), and negatively correlated with succinate (*p* = 0.01, *r* = −0.79), L-aspartic acid (*p* = 0.01, *r* = −0.78) and L-glutamic acid (*p* = 0.01, *r* = −0.78; Figure 10B).

Spearman correlation analysis was also performed on the differential metabolites related to fat metabolism with the differential gut microbiota in the BC and AG. Succinate were positively correlated with *Prevotella copri* (*p* = 0.03, *r* = 0.70), *Blautia wexlerae* (*p* = 0.02, *r* = 0.72) and *Ruminococcus gnavus* (*p* = 0.04, *r* = 0.67; Figure 10C). GABA were negatively correlated with *Prevotella copri* (*p* < 0.01, *r* = −0.84) and *Blautia wexlerae* (*p* < 0.01, *r* = −0.83; Figure 10C). Oxoglutaric acid were positively correlated with *Prevotella copri* (*p* < 0.01, *r* = 0.86), *Blautia wexlerae* (*p* = 0.02, *r* = 0.73) and *Ruminococcus gnavus* (*p* = 0.02, *r* = 0.75; Figure 10C). L-aspartic acid levels were positively correlated with *Prevotella copri* (*p* = 0.03, *r* = 0.70) and *Blautia wexlerae* (*p* = 0.03, *r* = 0.71; Figure 10C). L-asparagine levels were negatively correlated with *Prevotella copri* (*p* < 0.01, *r* = −0.84), *Blautia wexlerae* (*p* = 0.02, *r* = −0.73) and *Ruminococcus gnavus* (*p* = 0.03, *r* = −0.70; Figure 10C). L-glutamic acid levels were positively correlated with *Blautia wexlerae* (*p* = 0.01, *r* = 0.82) levels (Figure 10C). Fumaric acid were negatively correlated with *Prevotella copri* (*p* = 0.03, *r* = −0.71), *Blautia wexlerae* (*p* < 0.01, *r* = −0.83) and *Ruminococcus gnavus* (*p* = 0.01, *r* = −0.81; Figure 10C).

## Discussion

Our present study showed that the body weight, the carcass weight and the EMA, IMF and the IAA of Angus cattle were significantly higher than those of Xinjiang brown cattle, whereas the BFT and the MFSA of Xinjiang brown cattle were higher. The carcass weight, EMA, and the MFSA were indicators of carcass muscle strength and meat yield (Choi and Kim, 2009; Lang et al., 2016). The subcutaneous fat deposition is a necessary stage in the beef cattle fattening process. The intramuscular adipocytes are mainly derived from fibroblasts that reside in the connective tissue surrounding and within skeletal muscle (Lowe et al., 2012). The proportion of IMF content to total fat content is affected by variety, age, nutrition, and other factors (Bong et al., 2012; Gotoh and Joo, 2016). Tenderness, juiciness, flavor, and marbling all increase as IMF content increases (Farrow et al., 2009). Beef tenderness is improved by intramuscular adipose tissue infiltration, which destroys the collagen cross-linking that determines meat toughness (McEvers et al., 2012). Because adipose tissue is denser than muscle tissue, increased amounts of IMF also make lean meat taste more delicate and may help muscles retain more moisture (Campos et al., 2016). The results herein showed that the meat quality of Angus cattle is preferable than that of Xinjiang brown cattle.

Studies have shown that the gut microbiome is closely related to host macronutrient and lipid metabolism, thus affecting the meat quality traits of IMF content, BFT and tenderness. The gut microbiome may play a part in determining meat quality in cattle (Nicholson et al., 2012; Zhang et al., 2017). By analysis of the host-microbiota-metabolic axis revealed that gut microbiota is closely related to fat, sugar, and protein metabolism. Animal lipid metabolism-related meat quality traits are closely related to the composition of gut microbiota involved in lipid metabolism (Nicholson et al., 2012; Sun et al., 2016; Zhang et al., 2017). Zierer et al. (2018) analyzed the relationship between gut microbiota, host phenotype, and complex genetic traits in human twins and found that the microbiota structure is closely related to fat deposition. Gut microbiota imbalances or alterations in microbiota structure have also been shown to cause alterations in whole blood glucose and triglyceride levels, suggesting gut microbiota may regulate carbohydrate and lipid metabolism in the host body (Kuno et al., 2018). In our present study, the results of the α-diversity analysis showed that the richness and diversity of gut microbiota in Angus cattle were greater than those in Xinjiang brown cattle. Furthermore, results of LEfSe analysis showed Angus cattle possess a greater variety of microbiota species in their gut microbiota compared to Xinjiang brown cattle. Significant differences in microbiota composition were also observed following β diversity analysis. Previous studies have shown that obesity is related to the relative abundance of Bacteroidetes and Firmicutes or changes in the ratio of Firmicutes / Bacteroides win the gut microbiota (Turnbaugh et al., 2006; Parnell and Reimer, 2012). The ratio of Firmicutes / Bacteroides is related to the production of short-chain fatty acids. These acids serve as macromolecular substances that maintain host balance and disease and have been used to prevent or treat obesity and type 2 diabetes (Andrade-Oliveira et al., 2015; Bindels et al., 2015; Zhang et al., 2015; Ohira et al., 2017; Thursby and Juge, 2017). For example, An et al. (2018) showed the gut microbiota in high-fat diet-induced obese rats was composed of higher levels of Firmicutes and lower levels of Bacteroidetes. Increase of Bacteroidetes is related to increased production of short-chain fatty acids, which provide cellular energy, maintain the intestinal epithelial barrier, and regulate the immune system (Greenhalgh et al., 2016). Ley et al. (2005) found that obesity was related to an increase in the number of Firmicutes, a decrease in the number of Bacteroides, or an increase in the ratio of Firmicutes / Bacteroides in the gut. Bacteroidetes abundance is altered by environmental factors, such as dietary protein and fat content, and is also positively correlated with the deposition of animal fat (David et al., 2014; Johnson et al., 2017; Rampelli et al., 2018). Genus *Prevotella* is among the most abundant bacteria found in the rumen, they break down cellulose and use products of cellulose degradation as an energy source in sheep and cattle (Ellison et al., 2017; Delgado et al., 2019), *Prevotella copri* is ubiquitous in the intestine and considered a potential marker for distinguishing high feed efficiency in beef cattle during life span and production cycles (Brooke et al., 2019). A greater abundance of *Prevotella copri* in the gut microbiota is associated with abnormal carbohydrate metabolism during obesity (Duan et al., 2021), along with fat accumulation (Chen C. et al., 2021). Zheng et al. (2022) compared the gut microbiota and intramuscular differentially expressed genes in the Angus and Chinese Simmental cattle. The results show that the relative abundance of *Prevotella copri* was significantly higher in the Simmental cattle. *Blautia wexlerae* is yet another obesity-related bacterium. In contrast to *Prevotella copri*, *Blautia wexlerae* abundance is decreased during obesity (Benítez-Páez et al., 2020). An important function of *Blautia wexlerae* is to produce acetic acid and butyric acid (Jang et al., 2019). A decreased abundance of *Blautia wexlerae* leads to decrease in acetic acid and butyric acid levels (Vital et al., 2018). Our findings to some extents were supported by some recent publications. For example, Cao et al. (2021) fund that *Butyrivibrio*, *CF231* and *Dorea* were negative correlated with water loss of sheep meat quality traits significantly. Lei et al. (2021) showed that the higher relative abundances of the genera *Ruminococcaceae_NK4A214_group*, *Parabacteroides*, *Christensenellaaceae_R-7_group*, and *Ruminiclostridium* might corelate with higher intramuscular fat (IMF) content. Zheng et al. (2022) found that the relative abundance of *Roseburia*, *Prevotella*, and *Coprococcus* were positive correlated with IMF of beef quality traits significantly. Animal experiments with Khan and Jena (2016) showed that butyric acid may improve insulin sensitivity and reduce insulin resistance. Recent studies have found that the abundance of *Ruminococcus gnavus* in the intestine of ruminants was increased in obese animals, decreased with malnourishment, and positively correlates with body mass index (Blanton et al., 2016; Jang et al., 2019). In the present studies, the abundance of Bacteroidetes in the gut microbiota of Xinjiang brown cattle, i.e., *Prevotella*, *Blautia* at the genus level, *Prevotella copri*, *Blautia wexlerae*, and *Ruminococcus gnavus* at the species level were significantly lower than that of Angus cattle. Microbiota structure was related to differences in lipid metabolism, fat deposition, and obesity. Correlation analysis between *Prevotella, Blautia*, *Prevotella copri*, *Blautia wexlerae*, *Ruminococcus gnavus*, and meat quality indices related to lipid metabolism showed positive correlations with IMF amount and negative correlations with the BFT. These correlations suggest that *Prevotella*, *Blautia*, *Prevotella copri*, *Blautia wexlerae*, and *Ruminococcus gnavus* play an important role in regulating meat quality in Xinjiang brown cattle. Hosomi et al. (2022) performed a cross-sectional study of Japanese adults and identified that the *Blautia* genus, especially *Blautia wexlerae*, as a commensal bacterium inversely correlated with obesity and type 2 diabetes mellitus, furthermore, they administrated *Blautia wexlerae* orally to the high fat diet (HFD) fed mice, the body weight and the weight of epididymal adipose tissue were significantly decreased than those HFD mice without *Blautia wexlerae* administration demonstrating *Blautia wexlerae* has potential to contribute to the prevention of obesity. This research was partly supported our result that *Blautia wexlerae* was negative correlated with IMF. The correlation of MQT-LM and certain bacterial species showed that these species were mostly related to IMF, the BFT, the IAA, body weight, and carcass weight. Among them, *Prevotella* sp. *P3-122*, *Prevotella sp. P3-120*, *Prevotella sp. P2-180*, and *Prevotella sp. AM42-24* in the genus *Prevotella* play an important role in polysaccharide degradation and fermentation in the rumen (Shinkai et al., 2022). Zeybel et al. (2022) found that the abundances of *Prevotella* sp. *AM42 24* were significantly reduced in subjects with mild hepatic steatosis versus no hepatic steatosis. Eubacterium hallii affects the metabolic balance of the host by forming different short-chain fatty acids in the intestinal tract (Engels et al., 2016), and it was increased in small intestinal biopsies of obese and insulin-resistant subjects upon lean donor fecal transplantation associated with improved insulin sensitivity (Wan et al., 2019) suggesting it may be associated with MQT-LM. However, there is a lack of study on *Blautia sp. AF13-16* in association with lipid metabolism. These data were further supported by the KEGG and KO analyses which showed that lipid metabolism was different in *Prevotella intermedia* between Xinjiang brown and Angus cattle.

Gut microbiota interacts with its host, receiving energy from and providing energy to the host. This constant exchange of energy between microbiota and host is accomplished through the release of enzymes and metabolites such as short-chain fatty acids, amino acids, bile acids, casein B, and lipopolysaccharide (Fetissov, 2017). Importantly, *Escherichia coli* secretes casein B and casein B may enhance the peptide YY (PYY) and glucagon-like peptide (GLP) secretion (Breton et al., 2016). PPY and GLP decrease appetite, leading to decreased caloric intake and fat deposition (Murphy and Bloom, 2006; Berthoud, 2011; Zhang and van den Pol, 2017; Leidmaa et al., 2020). Fatty acids in food are absorbed by intestinal epithelial cells, where they are oxidized and degraded or re-esterified into triglycerides. Newly synthesized triglycerides are incorporated into cytoplasmic lipid droplets or chylous particles that are secreted in the lymphatic system (Iqbal and Hussain, 2009; Nakajima et al., 2014). The digestion of polysaccharides provides monosaccharides to intestinal epithelial cells and reduces the absorption of fatty acids, and promotes storage of newly synthesized triglycerides in cytoplasmic lipid droplets. *Escherichia coli* acts in conjunction with epithelial cells to form mono- and disaccharides that increase fatty acid absorption and oxidation. Increased fatty acid oxidation results in decreased triglyceride synthesis, chylous particle size, and chylous granule secretion, leading to the depletion of cytoplasmic lipid droplets (Poquet and Wooster, 2016; Tazi et al., 2018). Although it is clear that *Escherichia coli* plays a role in regulating lipid metabolism, but there is no research on the relationship between *Prevotella intermedia* and lipid metabolism. Functional level analysis of bovine gut microbiota in our present study showed that there were differences in the expression of pgpB and GPCPD1 genes within the “Glycerophospholipid metabolism” pathway in *Escherichia coli* and *Prevotella intermedia* suggesting that *Escherichia coli* and *Prevotella* are key bacteria that affect the MQT-LM in Xinjiang brown cattle.

Gut microbiota plays a role in maintaining host homeostasis and health *via* the production of short-chain fatty acids and neuroactive substances (Wang et al., 2020; Han et al., 2021). The neuroactive substance GABA is a major inhibitory neurotransmitter in the central nervous system (Cuevas-Sierra et al., 2021). GABA is also produced by intestinal microbiota to exhibit the largest change of metabolites in the intestine of obese patients in response to fecal transplant using samples from lean patients. This change of GABA was also related to the improvement in insulin sensitivity (Kootte et al., 2017). GABA may also play a role in the treatment of lipid metabolism disorders caused by type 1 and 2 diabetes by inducing pancreatic β-cells and stimulating insulin secretion (Soltani et al., 2011; Tian et al., 2011, 2013). Treatment of obese mice with metabolic dysfunction mice with GABA-producing bacteria attenuated the metabolic dysfunction and reduced mesenteric adipose tissue accumulation (Patterson et al., 2019). GABA can be metabolized to succinate, a metabolic by-product of anaerobic fermentation performed in Bacteroides (Zhang et al., 2008; Feehily and Karatzas, 2013; De Vadder et al., 2016; Louis and Flint, 2017). Interestingly, succinate inhibits fatty acid release from adipocytes. Patients with higher circulating succinate levels have higher blood glucose levels that may be related to changes in gut microbiota and increased barrier permeability. Thus, microbial-derived succinate may play an important role in obesity and metabolism-related cardiovascular disease (Serena et al., 2018). The oxoglutaric acid, succinate and fumaric acid are metabolites of the citric acid cycle pathway that link metabolic pathways and lead to the formation of citric acid (Akram, 2014). L-glutamic acid is an amino acid that can be utilized for carbohydrate production and is the precursor of glutathione. Meanwhile, L-glutamic acid is the first line of defense against free radicals in the liver and plays an essential role in the pathogenesis of metabolic diseases (Melis et al., 2004; Zhou et al., 2019). Glutamine was previously thought to be associated with obesity (Curtasu et al., 2019) and diabetes (Newgard et al., 2009), as higher levels of glutamine and related metabolites are part of the systematic response to higher blood glucose levels and act to stimulate insulin secretion to lower blood glucose levels (Newsholme et al., 2005). In addition, other studies have shown that plasma L-asparagine levels are negatively correlated with blood lipid levels and positively correlated with type 2 diabetes risk (Ottosson et al., 2018), and exogenous L-aspartic acid feeding limits fatty liver progression (Yanni et al., 2010). The results of the metabolomic analysis in the present study showed that oxoglutaric acid, succinate, fumaric acid, L-aspartic acid, L-asparagine, L-glutamic acid, and GABA levels differed between the two breeds of cattle, and differential metabolites were associated with MQT-LM. Specifically, IMF was positively correlated with succinate, oxoglutaric acid, and L-aspartic acid, and negatively correlated with GABA, L-asparagine, and fumaric acid. The BFT was positively correlated with GABA, L-asparagine, and fumaric acid, and negatively correlated with succinate, L-aspartic acid, and L-glutamic acid, suggesting that these metabolites may reflect alterations in lipid metabolism that determine meat quality. Associations between metabolomes, microbiota, and lipid metabolism functional expressions of the microbiota that differed between Xinjiang brown and Angus cattle showed that succinate, oxoglutaric acid, L-aspartate, and L-glutamic acid were positively correlated with the most abundant microbiota and that differed between breeds. Levels of GABA, L-asparagine, and fumaric acid were negatively correlated with the most abundant microbiota that was different between breeds, suggesting that MQT-LM may be regulated by changing the expression of lipid metabolism-related metabolites in gut microbiota. The correlation of microbiota and species (RA abv.0.1%) showed that except for the species correlation beef quality traits, there are four species of *Prevotella stercorea*, *Ruminococcaceae bacterium YAD3003*, *Clostridium* sp. *af27-5AA*, and *Acetivibrio ethanolgignens* related to lipid metabolites. Pisanu et al. (2020) evaluate the impact of nutritional intervention on the gut microbiota of obese and overweight patients, then patients presented a statistically significant reduction in body weight and fat mass and an increase in the abundance of *Prevotella stercorea*. Yuan et al. (2020) found that alteration of the gut microbiota *Acetivibrio ethanolgignens* under tributyltin exposure was involved in further mediating liver inflammation, causing lipid metabolism abnormalities with the energy supply process. Therefore, *Acetivibrio ethanolgignens* may be a candidate for a potential microbial community marker for assistant diagnosis. But more supporting study regarding the potentially useful markers of specific gut microbiota from meat animal origin are expected.

## Conclusion

There are significant differences in meat quality traits in association with gut microbiota and its lipid metabolism related metabolites between Xinjiang brown cattle and Angus cattle. The intramuscular fat content was positively correlated with Bacteria species of *Prevotella copri*, *Blautia wexlerae*, and *Ruminococcus gnavus*, and the metabolites succinate, oxoglutaric acid, L-aspartic acid and L-glutamic acid, while negatively with GABA, L-asparagine and fumaric acid. The backfat thickness was negatively correlated with *Blautia wexlerae* and the metabolites succinate, L-aspartic acid and L-glutamic acid, while positively with GABA, L-asparagine and fumaric acid. Furthermore *Prevotella Copri*, *Blautia wexlerae*, and *Ruminococcus gnavus* was all positively correlated with succinate, oxoglutaric acid, while negatively with L-asparagine and fumaric acid. Lipid metabolism genes within the glycerophospholipid metabolism pathway is enriched in *Prevotella intermedia* and *Escherichia coli*. Our data suggest that *Prevotella copri*, *Prevotella intermedia*, *Blautia wexlerae*, and *Ruminococcus gnavus* may serve as the potential differentiated bacterial species in association with meat quality traits related to the lipid metabolism *via* their metabolites of oxoglutaric acid, succinate, fumaric acid, L-aspartic acid, L-asparagine, L-glutamic acid and GABA may serve as potential biomarkers to assistant for improving the meat quality in Xinjiang brown cattle.

## Data availability statement

The datasets presented in this study can be found in online repositories. The names of the repository/repositories and accession number(s) can be found at: https://www.ncbi.nlm.nih.gov/, PRJNA813004.

## Ethics statement

The animal study was reviewed and approved by the Animal Ethics Committee of Xinjiang Agricultural University (2017015).

## Author contributions

ZC and GY designed the study and wrote the manuscript. ZC, HL, and XY are responsible for field survey and experimental determination. ZC, JW, YZ, and LX are responsible for analyzed the data. ZC drafted the manuscript. GY revised the manuscript and approved final version of the manuscript. All authors contributed to the article and approved the submitted version.

## Funding

This work was supported by the Autonomous Region Natural Science Foundation of Xinjiang (2019D01A58), National Natural Science Foundation of China (31460647), and the Science and Technology Research Key Project in Department of Education, Xinjiang (2017B01001-2).

## Conflict of interest

The authors declare that the research was conducted in the absence of any commercial or financial relationships that could be construed as a potential conflict of interest.

## Publisher’s note

All claims expressed in this article are solely those of the authors and do not necessarily represent those of their affiliated organizations, or those of the publisher, the editors and the reviewers. Any product that may be evaluated in this article, or claim that may be made by its manufacturer, is not guaranteed or endorsed by the publisher.

## Supplementary material

SUPPLEMENTARY FIGURE S1 KEGG pathways enriched in Xinjiang Brown and Angus cattle microbiomes.

SUPPLEMENTARY FIGURE S2 Classification of differential metabolites.

SUPPLEMENTARY FIGURE S3 Metabolic pathway impact analysis. Alanine, aspartate and glutamate metabolism (-log(p) 4.52; Impact 0.63) (A); D-Glutamine and D-glutamate metabolism (-log(p) 1.89; Impact 1.00).

SUPPLEMENTARY FIGURE S4 Correlations of the species (RA abv 0.1%) - MQT-LMs - metabolites.
