# Supplementary figures and images for: Differences in meat quality between Angus cattle and Xinjiang brown cattle in association with gut microbiota and its lipid metabolism
Source: Front Microbiol. 2022 Dec 6;13:988984. doi: 10.3389/fmicb.2022.988984 (PMC9763702; doi:10.3389/fmicb.2022.988984)

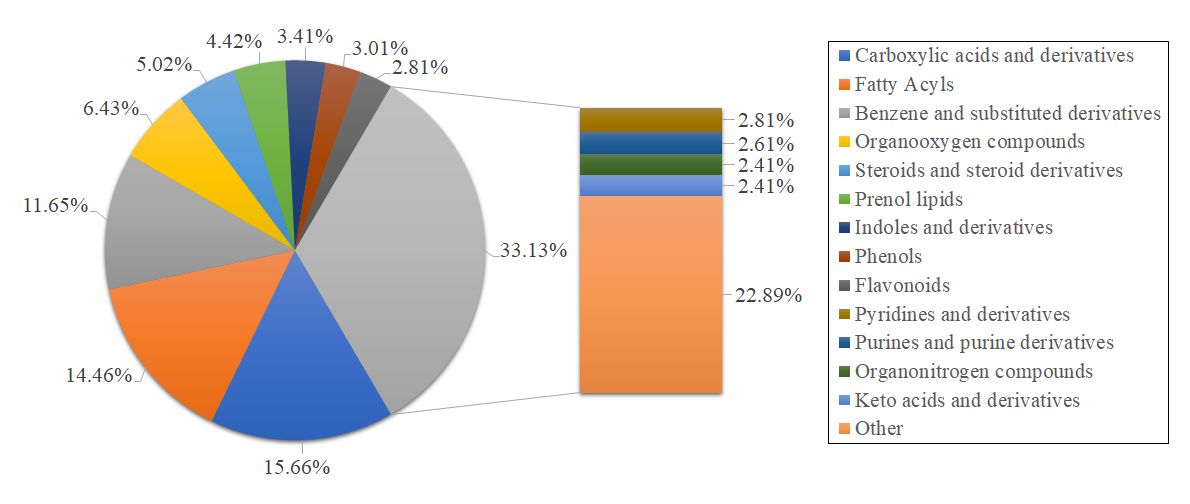

Supplement: SUPPLEMENTARY FIGURE S2 — Classification of differential metabolites. [file Image_2.TIF]

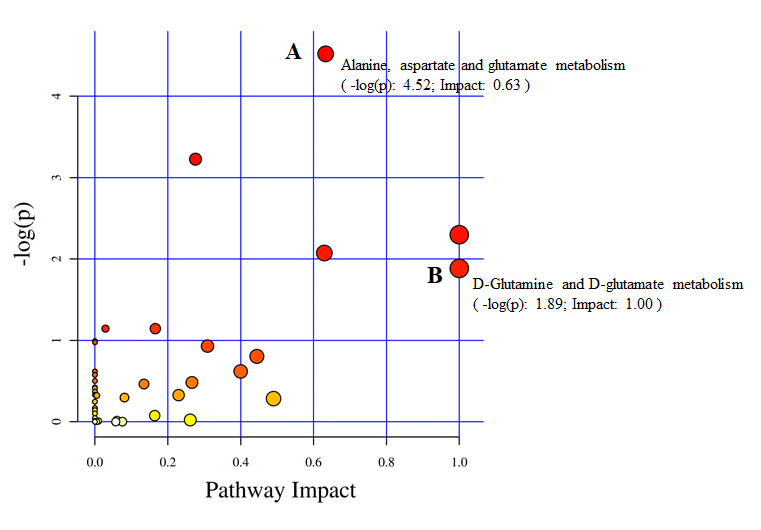

Supplement: SUPPLEMENTARY FIGURE S3 — Metabolic pathway impact analysis. Alanine, aspartate and glutamate metabolism (-log(p) 4.52; Impact 0.63) (A); D-Glutamine and D-glutamate metabolism (-log(p) 1.89; Impact 1.00). [file Image_3.TIF]

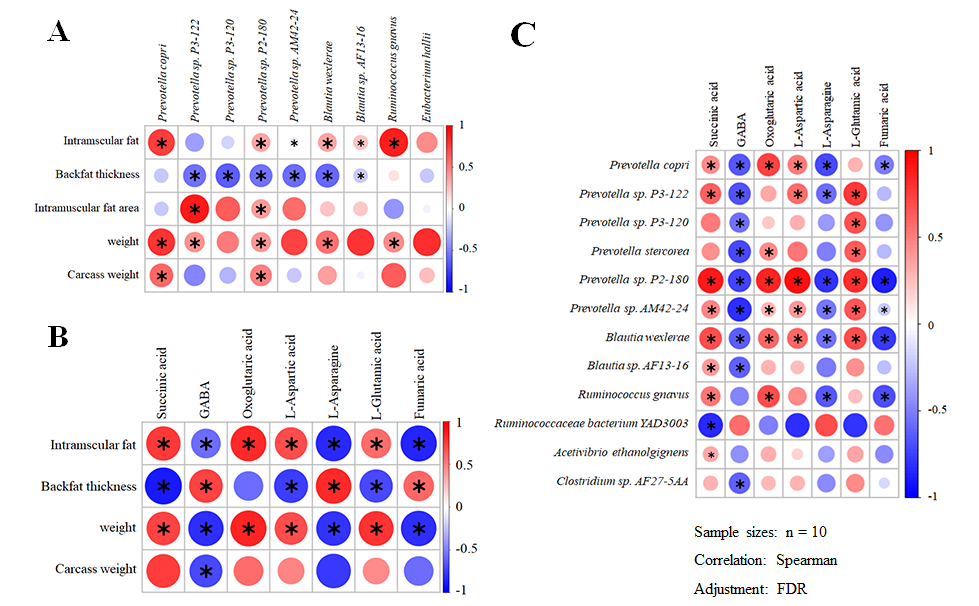

Supplement: SUPPLEMENTARY FIGURE S4 — Correlations of the species (RA abv 0.1%) - MQT-LMs - metabolites. [file Image_4.TIF]
